# Diagnostic Process Using Endoscopy for Biliary Strictures: A Narrative Review

**DOI:** 10.3390/jcm10051048

**Published:** 2021-03-03

**Authors:** Yuki Tanisaka, Masafumi Mizuide, Akashi Fujita, Tomoya Ogawa, Masahiro Suzuki, Hiromune Katsuda, Youichi Saito, Kazuya Miyaguchi, Tomoaki Tashima, Yumi Mashimo, Shomei Ryozawa

**Affiliations:** Department of Gastroenterology, Saitama Medical University International Medical Center, 1397-1, Yamane, Hidaka, Saitama 350-1298, Japan; mizuide1971@yahoo.co.jp (M.M.); a.fujita0628@gmail.com (A.F.); t.ogawa0210@icloud.com (T.O.); msuzgast@tmd.ac.jp (M.S.); hk0112@saitama-med.ac.jp (H.K.); stm_ys41@yahoo.co.jp (Y.S.); kaz.hr77@gmail.com (K.M.); tomo3029@saitama-med.ac.jp (T.T.); ymashimo@saitama-med.ac.jp (Y.M.); ryozawa@saitama-med.ac.jp (S.R.)

**Keywords:** biliary strictures, endoscopic retrograde cholangiopancreatography, ERCP, cholangioscopy, confocal laser endomicroscopy, endoscopic ultrasound, EUS, EUS-FNA

## Abstract

The diagnostic process for biliary strictures remains challenging in some cases. A broad differential diagnosis exists for indeterminate biliary strictures, including benign or malignant lesions. The diagnosis of indeterminate biliary strictures requires a combination of physical examination, laboratory testing, imaging modalities, and endoscopic procedures. Despite the progress of less invasive imaging modalities such as transabdominal ultrasonography, computed tomography, and magnetic resonance imaging, endoscopy plays an essential role in the accurate diagnosis, including the histological diagnosis. Imaging findings and brush cytology and/or forceps biopsy under fluoroscopic guidance with endoscopic retrograde cholangiopancreatography (ERCP) are widely used as the gold standard for the diagnosis of biliary strictures. However, ERCP cannot provide an intraluminal view of the biliary lesion, and its outcomes are not satisfactory. Recently, peroral cholangioscopy, confocal laser endomicroscopy, endoscopic ultrasound (EUS), and EUS-guided fine-needle aspiration have been reported as useful for indeterminate biliary strictures. Appropriate endoscopic modalities need to be selected according to the patient’s condition, the lesion, and the expertise of the endoscopist. The aim of this review article is to discuss the diagnostic process for indeterminate biliary strictures using endoscopy.

## 1. Introduction

Biliary strictures can lead to hepatobiliary dysfunction and eventually liver failure. They need to be appropriately treated, for example with biliary drainage and surgery; therefore, a correct diagnosis is necessary before treatment. A broad differential diagnosis exists between benign (including inflammatory) and malignant conditions. Benign biliary strictures are caused by primary sclerosing cholangitis (PSC), IgG4-related sclerosing cholangitis, bile duct stones, infection, ischemia related to surgical interventions, or iatrogenic injury. Malignant biliary strictures are caused by carcinomas involving the intra/extrahepatic bile duct, gallbladder (cystic duct), pancreatic duct, ampulla of Vater, liver, and metastatic cancers. Malignant biliary diseases have a poor prognosis, with an overall five-year survival as low as approximately 10%. The natural prognosis of cholangiocarcinoma without chemotherapy leads to an overall survival of 3.9 months. If palliative chemotherapy is used, median survival can be prolonged to 12–15 months [1,2]. If it can be treated surgically with R0 resection, long-term survival would be expected [3]. Therefore, correct and early diagnoses are required. Staging diagnosis and extension of the lesion are required to select a treatment or resection area. However, differentiating between benign and malignant bile duct strictures remains challenging in some cases. Multidisciplinary approaches such as physical examination, laboratory testing, imaging modalities, and endoscopic procedures are required to make a correct diagnosis. Despite the progress of less invasive imaging modalities such as transabdominal ultrasonography (AUS), computed tomography (CT), and magnetic resonance imaging (MRI), endoscopy plays an essential role in the accurate diagnosis, including the histological diagnosis. In this review, we discuss the diagnostic process using endoscopy for indeterminate biliary strictures.

## 2. Noninvasive Evaluation

Before performing endoscopic procedures, noninvasive evaluation is the first approach to diagnosing biliary strictures. It is important to take patient’s history in detail. Surgical operation history such as cholecystectomy, hepatectomy, or pancreatoduodenectomy has the potential to cause biliary strictures or anastomotic strictures. These strictures need to be treated by balloon dilation or stent placement [4]. Chronic pancreatitis could cause distal biliary stricture so it is necessary to examine the patient’s pancreatitis history or alcohol intake. He strictures related to PSC can be seen in chronic liver disorder or inflammatory bowel disease, so it is necessary to examine patients’ inflammatory bowel disease history. Moreover, PSC patients could be concurrent with cholangiocarcinoma in 10–15% of cases [5]. Therefore, careful follow-up for PSC patients is required. Besides the patient’s history, it is also important to examine the patient’s symptoms, such as jaundice, abdominal pain, fever, lymphadenopathy, and decreased blood pressure. These symptoms could be detected patients with biliary strictures.

Laboratory testing is performed following taking the patient’s history and examining the patient’s symptoms. Hepatobiliary dysfunction is detected in cases of biliary strictures. The higher the bilirubin level, the more likely that the stricture is malignant [6]. IgG4 levels are examined for autoimmune pancreatitis and IgG4-associated cholangitis but may also be elevated in cholangiocarcinoma and PSC [7,8,9]. Tumor markers provide better insight into the presence of malignancies that cause biliary stricture. Serum carbohydrate antigen (CA) 19-9 is a tumor marker that is useful for pancreatobiliary cancer with a sensitivity of approximately 70% for pancreatic cancer and 50–80% for bile duct cancer [10,11]. However, CA19-9 increases in the presence of jaundice, and false positives may arise in cases of biliary strictures. Moreover, one study reported although CA19-9 had a sensitivity and specificity of 79–81% and 82–90%, respectively, for the diagnosis of cancer, it was not so useful as a screening marker because of its low positive predictive value (0.5–0.9%) [12]. Carcinoembryonic antigen (CEA) has a sensitivity and specificity of 53–84% and 50–79%, respectively, for cholangiocarcinoma [13]. Although tumor markers are good to examine as an initial laboratory test, they are insufficient to make a correct diagnosis so other modalities should be added.

Cross-sectional imaging is performed to detect lesions that cause biliary strictures. AUS is frequently used as a first-line imaging test, and it may suggest the location of a biliary stricture, although a detailed anatomic description is usually difficult. The sensitivities of AUS for detecting hilar bile duct cancer, middle, and distal were 86, 59, and 33%, respectively [14]. Bile duct dilatation on AUS findings is an important sign for the early diagnosis of bile duct cancer. Both CT and MRI have high diagnostic accuracy for the identification and characterization of primary lesions [15,16] and to determine the resectability of malignancies [16,17]. Therefore, multiphasic contrast-enhanced CT or MRI should be considered in the initial evaluation of patients with biliary strictures prior to endoscopic procedures. CT and MRI have the added advantage of potentially detecting distant metastasis. Both CT and MRI have high diagnostic accuracy for the identification and characterization of primary lesions so it is difficult to mention which modality is superior or not. Magnetic resonance cholangiopancreatography (MRCP) generally provides superior visualization of the bile duct, particularly with regard to the intrahepatic ducts, compared to CT [18]. Moreover, MRCP has the benefit of allowing for imaging of the proximal bile duct in cases of severe obstruction that may prevent contrast from traversing the stricture and/or preventing undrained areas during endoscopic retrograde cholangiopancreatography (ERCP) [19].

## 3. Endoscopic Retrograde Cholangiopancreatography with Cytology and Forceps Biopsy

**Figure 1:**
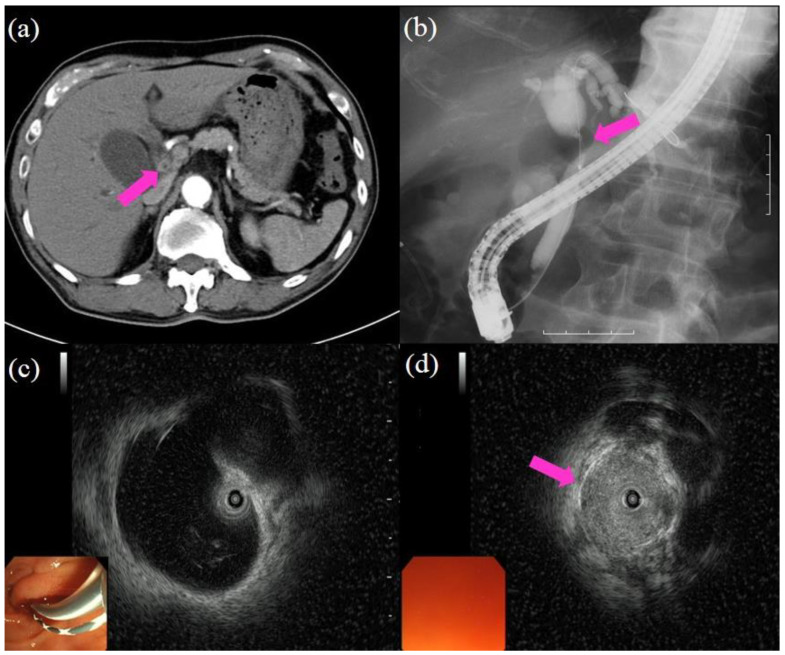
Procedures of cholangiography and intraductal ultrasound (IDUS): (**a**) computed tomography showing the wall thickness in the bile duct (pink arrow); (**b**) cholangiography showing the biliary stricture in the hilar bile duct (pink arrow); the proximal part of the bile duct shows dilatation; (**c**) IDUS showing dilatation in the proximal part of the bile duct and no lesion; (**d**) IDUS showing a lesion in the biliary stricture (pink arrow).

Since its introduction in 1968, endoscopic retrograde cholangiopancreatography (ERCP) has been an essential and established procedure for the diagnosis and treatment of biliopancreatic diseases. The success rate of the procedure has been reported to be approximately 95% [20,21], and it is still considered the gold standard for biliary imaging. A duodenoscope is advanced to the ampulla, and an ERCP catheter is inserted into the biliary tract over a guidewire. Following cannulation of the biliary tract, the contrast medium is injected for cholangiography. When performing ERCP, the interpretation of the cholangiography findings is the first step. An accurate distinction between benign and malignant biliary strictures is needed. Malignancy is suggested when the cholangiography shows strictures that are longer than 10 mm, asymmetric, and irregular. Benign disease is suggested when cholangiography shows short, regular, and symmetric strictures. Using these criteria, the diagnostic sensitivity and specificity for cholangiography findings were reported to be 74% and 70%, respectively [22]. After cholangiography, intraductal ultrasound (IDUS) is performed to detect the main lesion. Moreover, superficial extension from the main lesion or vascular invasion could be confirmed using IDUS [23,24,25]. A large retrospective study reported a sensitivity of 93.2%, a specificity of 89.5%, and an accuracy of 91.4% for the evaluation of malignant strictures [26]. When inserting the IDUS catheter into the bile duct, some cases are difficult due to the tension in the sphincter of Oddi. In such cases, endoscopic sphincterotomy may be performed. When inserting the IDUS catheter over the stricture, balloon dilation may be performed to pass the stricture. However, it should be limited for mandatory cases where investigation of proximal superficial extension is required because it might damage the main lesion. Figure 1 shows the procedures of cholangiography and IDUS.

Although cholangiography or IDUS findings provide information on whether the biliary stricture is benign or malignant, it is difficult to make a final diagnosis using only these methodologies. To make the final diagnosis, cytology/forceps biopsy under fluoroscopic guidance with ERCP is still the gold standard. Obtaining a specimen of adequate cellularity is essential for the evaluation of any potential malignancy. There are three approaches during ERCP: (1) bile juice aspiration cytology, (2) brush cytology, and (3) forceps biopsy.

Bile juice aspiration cytology is performed after the insertion of a biliary catheter. Although it is the easiest way to obtain a specimen, the sensitivity is extremely low, between 6% and 24% [27,28].

Regarding cytology, brush cytology may be considered a superior method to obtain a specimen compared to bile juice aspiration cytology. Brushing to obtain cytologic material involves advancing a brush with its catheter sheath through the endoscope into the biliary tree, generally over the guidewire. The device is advanced to the proximal part of the stricture, then the brush is advanced from the catheter, withdrawn slightly, and moved back and forth across the stricture approximately 15 times. The brush is then withdrawn into the catheter, and the device is withdrawn from the endoscope. The brush can be smeared onto glass slides, cut off from the device and placed into a fixative solution, or both. Its diagnostic performance has been evaluated in many studies with the sensitivity for malignancy in the range of 21–70% and specificity of 97–100% [29,30,31,32,33,34,35,36,37]. Table 1 summarizes the diagnostic yield of previous studies on brush cytology.

Forceps biopsy is more time consuming and more technically challenging than brush cytology because it is sometimes difficult to insert thick forceps into the bile duct and grasp a targeted specimen. However, it could provide a sample of bile duct tissue from deep in the epithelium, which is expected to improve diagnostic yield compared with brush cytology. The biopsy forceps are thicker than an ERCP catheter so it could be difficult to insert them into the bile duct. Difficult cannulation has been identified as a risk factor of post-ERCP pancreatitis. Therefore, it may be better to perform sphincterotomy in advance to facilitate biliary cannulation using biopsy forceps to prevent post-ERCP pancreatitis. Under fluoroscopic guidance, the forceps are advanced to the part of the stricture, opened, and then closed to grasp a specimen from the distal aspect of the stricture. Some reports suggested that at least three specimens should be obtained [30,38]. The diagnostic performance of forceps biopsy has also been evaluated in many studies, with the sensitivity for malignancy in the range of 43–81% and specificity, 90–100% [30,33,34,35,36,39,40,41]. Table 2 summarizes the data of previous studies on forceps biopsy under fluoroscopic guidance. A meta-analysis reported that the pooled sensitivity and specificity of the brush cytology for the diagnosis of biliary strictures was 45% (95% confidence interval (CI) (40–50%)) and 99% (95% CI (98–100%)), respectively [42], whereas forceps biopsy had a pooled sensitivity and specificity of 48.1% (95% CI (42.8–53.4%)) and 99.2% (95% CI (97.6–99.8%)), respectively. Although forceps biopsy may have better sensitivity than brush cytology, these results have an insurmountable limit under fluoroscopic guidance.

In the recent European Society of Gastrointestinal Endoscopy Guidelines [43], the rates of pancreatitis, cholangitis, and perforation during/post-ERCP have been reported to be 3.5–9.7%, 0.5–3.0%, and 0.08–0.6%, respectively [44,45,46]. Moreover, the mortality rate of post-ERCP pancreatitis has been reported to be 0.1–0.7% [44]. Although ERCP is an essential procedure to assess biliary strictures, we must be mindful that severe and fatal ERCP-related adverse events can occur.

## 4. Cholangioscopy

As described above, ERCP is the gold standard for diagnosing biliary strictures. However, ERCP does not provide an intraluminal view of biliary strictures. Cholangio/pancreatoscopy overcomes this limitation by allowing direct visualization of the biliary and pancreatic ducts. Moreover, it can perform targeted biopsies of the site of interest. The traditional “mother–daughter” peroral cholangioscopy (POCS) requires two endoscopists, with one operating the cholangioscope, while the second endoscopist controls the duodenoscope [47]. The limitations of this system are the need for two operators, scope fragility, and time consumption. Over the past decade, single-operator cholangioscopy (SOC) (SpyGlass™ Direct Visualization System, Boston Scientific, Marlborough, MA, USA) has been widely utilized with disposable fiberoptic technology [48]. The setup of SOC is easy; only one operator is needed, four-way tip deflection is allowed, and targeted biopsies and therapeutic procedures such as lithotripsy can be performed. Nowadays, the new digital SOC with high-resolution digital technology (SpyGlass DS Direct Visualization System) provides improved image quality and maneuverability of the catheter tip [49]. The system consists of a 10.8-Fr catheter. The POCS is generally advanced over a guidewire into the bile duct through the working channel of a duodenoscope. Before insertion, sphincterotomy is generally performed. The working channel (1.2-mm diameter in SOC) allows the passage of accessory devices and aspiration.

**Figure 2:**
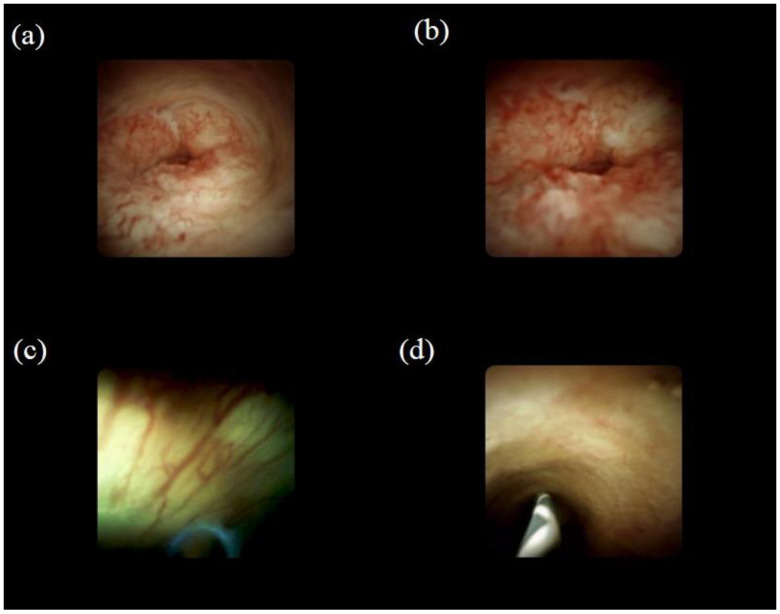
Visual findings of cholangioscopy: (**a**) irregular thick tortuous vessels, suggesting malignancy; (**b**) irregular papillogranular surface, suggesting malignancy; (**c**) fine network of thin vessels, suggesting a benign lesion; (**d**) lower homogeneous papillogranular surface without primary masses, suggesting a benign lesion.

POCS findings are defined as either malignant or benign according to the previous reports (Figure 2) [50,51,52,53]. Malignant findings include: (i) irregular thick tortuous vessels, (ii) oozing, (iii) irregular papillogranular surface, and (iv) a nodular elevated surface such as a submucosal tumor. Benign findings include: (i) a fine network of thin vessels and a flat surface with or without shallow pseudodiverticula; (ii) a lower homogeneous papillogranular surface without primary masses, suggesting hyperplasia; (iii) a bumpy surface with or without pseudodiverticula, suggesting inflammation; and (iv) a white surface with a convergence of folds, suggesting scarring. Table 3 provides the data on the diagnostic yield of POCS visual findings for indeterminate biliary strictures [37,41,48,49,54,55,56,57,58]. The sensitivity for malignancy is in the range of 83–100%, the specificity is 67–96%, and the accuracy is 85–96%. In a systematic review and meta-analysis of the diagnostic yield of POCS visual findings, the pooled sensitivity and specificity for diagnosing malignant biliary strictures were 84.5% (95% CI (79.2–88.9%)) and 82.6% (95% CI (77.1–87.3%)), respectively [59]. Moreover, POCS can detect superficial extension of cholangiocarcinoma in detail. It has been reported that POCS-guided biopsy provides an accurate diagnosis of the superficial extension of the cholangiocarcinoma compared with ERCP alone [60].

Despite good outcomes, the visual criteria for malignancy are not yet fully established, and there is significant interobserver variation in the interpretation of POCS visual findings. These variations can be misleading and may result in false-positive malignant diagnoses. Therefore, definite pathological confirmation is important for a definitive diagnosis of indeterminate biliary strictures. A prospective study reported that POCS-guided 3Fr mini-forceps tissue sampling has significantly higher accuracy compared with fluoroscopy-guided standard forceps biopsies [61]. Table 3 also provides data on the diagnostic yield of POCS-guided biopsy for indeterminate biliary strictures. The sensitivity for malignancy is in the range of 64–86%, the specificity is 89–100%, and the accuracy is 70–90% [37,41,48,49,54,55,56,57,58]. In a systematic review and meta-analysis of the diagnostic yield of the POCS-guided biopsy, the pooled sensitivity and specificity for diagnosing malignant biliary strictures were 60.1% (95% CI (54.9–65.2%)) and 98.0% (95% CI (96.0–99.0%)), respectively [59].

**Figure 3:**
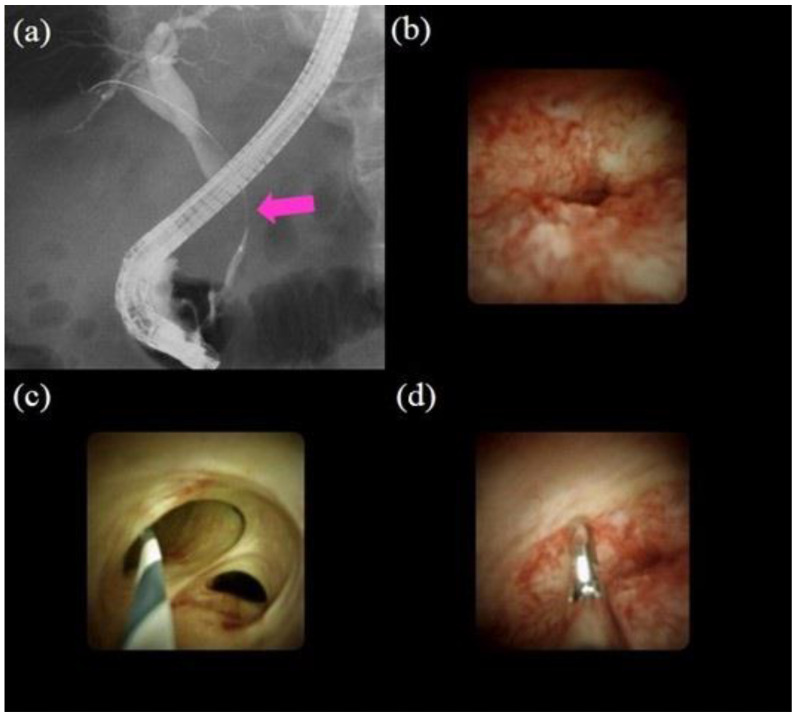
Procedures of peroral cholangioscopy (POCS) and biopsy under direct view with POCS: (**a**) cholangiography showing the biliary stricture in the distal bile duct (pink arrow); (**b**) POCS showing an irregular papillogranular surface at the stricture, suggestive of malignancy; (**c**) POCS showing a fine network of thin vessels at the hilar bile duct, suggesting no malignancy; (**d**) forceps biopsy under direct view with POCS; the histological examination revealed adenocarcinoma.

In a meta-analysis regarding POCS procedures, overall and serious adverse event rates of 7% and 1%, respectively, were reported [62]. When performing POCS, we must be mindful that cholangitis could be caused by an increase in intraductal pressure due to water irrigation during the procedure. Therefore, it is necessary to use antibiotic prophylaxis and perform biliary drainage to prevent cholangitis. Figure 3 highlights the procedure using POCS (SOC) to diagnose biliary strictures.

## 5. Confocal Laser Endomicroscopy

Confocal laser endomicroscopy (CLE) is an endoscopic imaging technique that can provide in vivo histological assessment in real-time, known as “virtual biopsy.” Probe-based CLE (pCLE; CholangioFlex, Cellvizio; Mauna Kea Technologies, Paris, France) has been cited in the recent American Society for Gastrointestinal Endoscopy guidelines for the management of biliary neoplasia as a useful alternative to the existing diagnostic workup [63]. pCLE is performed under fluoroscopy guidance or direct view with POCS during ERCP. The CholangioFlex pCLE probe is designed to obtain in vivo, real-time, microscopic images of the bile duct during ERCP procedures. The probe has a diameter of 0.94 mm, a field of view of 325 µm, and a lateral resolution of 3.5 µm. Each probe provides images from 40–70 µm below the tissue surface. The confocal probe is advanced through the working channel of the POCS and gently applied to the part of interest to carry out confocal imaging at 12 frames per second. Intraductal images are recorded and saved to a computer unit connected to the probe. Although pCLE can be performed both under fluoroscopy guidance or direct view with POCS, the pCLE findings under direct view with POCS can be accurately matched with biopsy tissue. Therefore, these results could be diagnostically more reliable [58,64].

**Figure 4:**
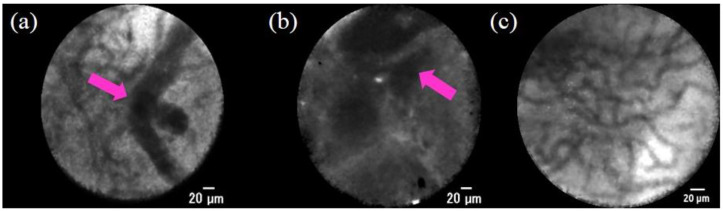
Probe-based confocal laser endomicroscopy images for biliary strictures: (**a**) thick dark bands (>40 µm) (pink arrow) according to the Miami classification; (**b**) dark clumps (pink arrow) according to the Miami classification; (**c**) reticular network of thin dark branching bands (<20 µm) according to the Miami classification.

The Miami classification was initially created to differentiate malignant and benign tumors [65] (Figure 4). The criteria for the diagnosis of malignancy are listed as follows: (1) thick white bands (>20 µm), (2) thick dark bands (>40 µm), (3) dark clumps, and (4) epithelium. The criteria for the diagnosis of benign lesions are as follows: (1) a reticular network of thin dark branching bands (<20 µm), (2) a light-gray background, and (3) blood vessels (<20 µm). Subsequently, to solve the problem of false-positive cases (such as inflammation) as a result of using the Miami classification, the Paris classification was created [66].

**Figure 5:**
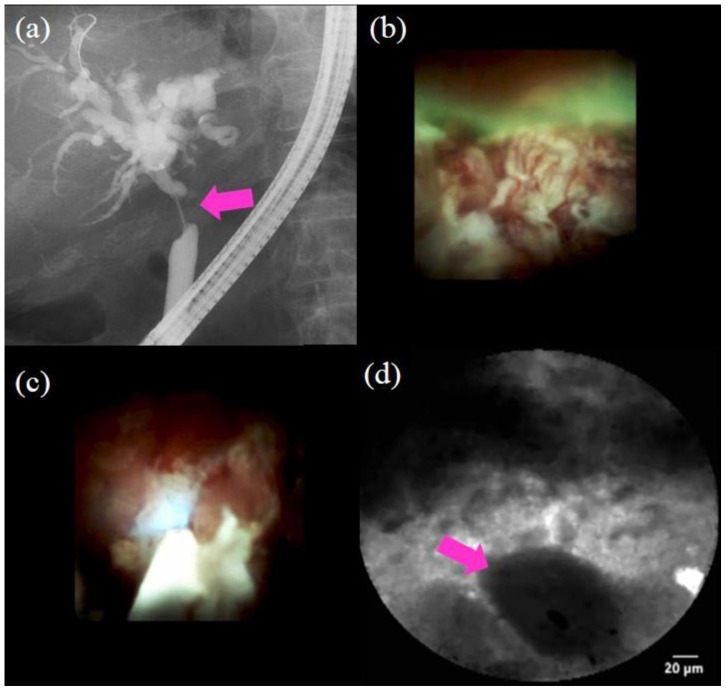
Procedure of probe-based confocal laser endomicroscopy (pCLE) under direct view with peroral cholangioscopy (POCS) to diagnose biliary strictures: (**a**) cholangiography showing the biliary stricture in the hilar bile duct (pink arrow); (**b**) POCS showing irregular thick tortuous vessels at the stricture, suggestive of malignancy; (**c**,**d**) pCLE under direct view with POCS showing dark clumps, suggestive of malignancy; the histological examination demonstrated adenocarcinoma.

Table 4 provides the data on the diagnostic yield of pCLE for biliary strictures [58,64,65,66,67,68]. The sensitivity for malignancy is in the range of 83–98%, the specificity is 33–93%, and the accuracy is 78–93%. In a systematic review and meta-analysis of the diagnostic yield of pCLE, the pooled sensitivity and specificity for diagnosing malignancy were 90% (95% CI (84–94%)) and 75% (95% CI (66–83%)), respectively [69]. Figure 5 highlights the procedure of pCLE under direct view with POCS to diagnose biliary strictures. pCLE has been shown to have high-performance characteristics in the evaluation of biliary strictures, possibly reducing the need for repeat procedures, thereby decreasing cost. However, CLE requires additional training for interpretation. This variability of interpretation is considered to be the greatest obstacle to the widespread use of CLE.

## 6. Endoscopic Ultrasound and Endoscopic Ultrasound-Guided Fine-Needle Aspiration

Endoscopic ultrasound (EUS) is an ultrasound technique in which the tip of the endoscope is equipped with a high-frequency transducer. High-resolution images of the biliary tract can be obtained through the stomach and duodenum. Regarding malignant biliary stricture detection, EUS without fine-needle aspiration (FNA) was found to provide a sensitivity of 78% and specificity of 84% [70]. Another study proved that EUS was superior for the detection of malignancies compared to CT and MRI (94, 30, and 42%, respectively) [71]. Regarding adverse events, EUS, especially for observation purposes, can avoid pancreatitis, which is mainly problematic for ERCP.

EUS-FNA is the established diagnostic modality to obtain specimens, particularly of pancreatic tumors [72,73]. EUS-FNA enables the acquisition of histological evidence of cancer when chemotherapy is being considered to distinguish benign or malignant tumors when deciding whether surgery or follow-up is needed, and assessment of the degree of progression of malignant tumors when unexplained lymph node swelling is detected. At present, the most frequently used needle sizes are 22 gauge and 25 gauge. Table 5 provides the data on the diagnostic yield of EUS-FNA of the biliary tract [71,74,75,76,77,78]. The sensitivity for malignancy is in the range of 43–94%, the specificity is 100%, and the accuracy is 70–94%. In a recent meta-analysis, it was reported that the mean sensitivities of ERCP and EUS-FNA for the diagnosis of malignant biliary strictures were 49% and 75%, while specificities were 96% and 100%, respectively [79]. EUS-FNA might offer a safer alternative to ERCP. With the recent progress of needles, the fine-needle biopsy (FNB) device, which was designed primarily to obtain core tissue samples, was introduced to overcome the FNA sampling material limitation [80,81]. In a recent meta-analysis comparing FNA with FNB needles, FNB provided a higher pooled diagnostic accuracy, tissue core rate, and allowed diagnosis with fewer passes in both pancreatic and nonpancreatic lesions [82]. Although there were no reports using FNB needles regarding the biliary tract, FNB needles have the potential to increase the diagnostic accuracy. Hence, studies regarding EUS-FNB use for the biliary tract are warranted. Recently, increasing case reports of needle tract seeding following EUS-FNA or EUS-FNB are emerging. In a recent review regarding needle tract seeding following EUS-FNA or EUS-FNB, 33 patients (27, pancreatic cancer; 6, others) with needle tract seeding following EUS-FNA or EUS-FNB have been reported up to January 2020 [83]. Although there were no reports regarding the biliary tract, needle tract seeding could be caused. Thus, EUS-FNA should not be performed when it does not guide treatment selection [84].

## 7. Molecular Diagnostics

Next-generation DNA sequencing (NGS) as molecular diagnostics has been the upcoming technology for diagnosing biliary strictures. It allows for the rapid and simultaneous sequencing of genetic material on a single medium or surface [85,86]. One study showed that the combination of a 28-gene panel (BiliSeq) and pathological evaluation increased the sensitivity to 83% and the specificity of 99% in diagnosing biliary strictures [86]. Although further studies are required, it has the potential to diagnose biliary strictures and identify targetable genomic alterations.

## 8. Percutaneous Transhepatic Cholangiography

Percutaneous Transhepatic Cholangiography (PTC) is a radiologic procedure that directly accesses the biliary tract using an ultrasound-guided percutaneous needle. It was reported that the sensitivity and specificity of PTC were 70.8% and 47.6%, respectively [87]. It is similar diagnostic ability to ERCP. In this study, percutaneous transhepatic cholangioscopy (PTCS) was also useful for diagnosing biliary strictures. Although PTC is a useful option to diagnose biliary strictures, seeding could be caused by PTC. In a recent systematic review comparing the incidence of seeding metastasis between endoscopic biliary drainage (EBD) and PTC reported that the incidence of seeding metastasis in the EBD group was significantly lower than that in the PTBD group (10.5% vs. 22.0% OR = 0.35, 95% CI 0.23~0.53) [88]. Therefore, PTC might be limited to cases in which ERCP-related procedures have failed.

## 9. Conclusions

**Figure 6:**
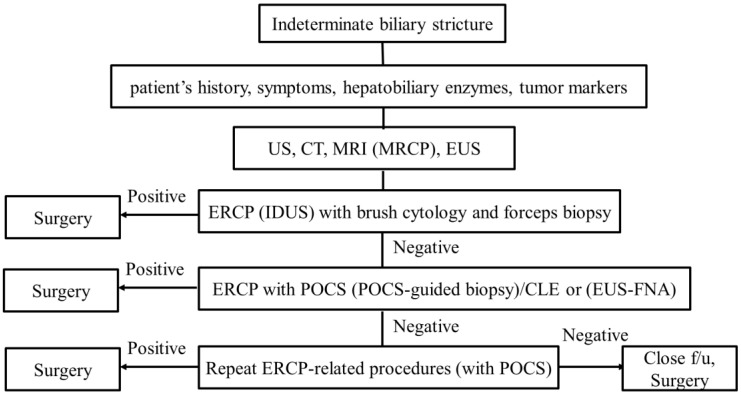
Diagnostic algorithm for indeterminate biliary strictures.

We discussed the diagnostic process using endoscopy for indeterminate biliary strictures. Various modalities using endoscopy for the diagnosis of biliary strictures have been reported, and their capabilities have improved. We propose the diagnostic algorithm (Figure 6). First of all, noninvasive evaluation such as taking the patient’s history, examining the patient’s symptoms, hepatobiliary enzymes, and tumor markers should be performed. Second, cross-sectional imaging such as US, CT, and MRI (MRCP) should be performed. EUS imaging is also useful at the same time. Third, an ERCP-related procedure should be performed. As we showed, peroral cholangioscopy (POCS) findings and POCS-guided biopsy/Confocal laser endomicroscopy (CLE) provide better outcomes than ERCP under fluoroscopic guidance. However, as POCS and CLE are too expensive to use in the first instance, ERCP (IDUS) with brush cytology and forceps biopsy should be performed first. If the ERCP with brush cytology and forceps biopsy is positive, surgery should be performed. If the stricture remains indeterminate, ERCP with POCS (POCS-guided biopsy)/CLE should be performed. Although EUS-FNA may be performed at this time, we must take into consideration that seeding could be caused. If the stricture remains indeterminate, repeat consideration should be made for repeat ERCP with brushings, POCS with biopsies, and pCLE. If the stricture remains indeterminate even though repeat procedures were performed and suspicion for malignancy remains high, close follow-up or surgery might be considered. Although progress has been made regarding endoscopic procedures, further improvement is needed.

## Figures and Tables

**Table 1:** The diagnostic yield of studies on brush cytology.

Authors	Year	Number of Patients	Sensitivity (%)	Specificity (%)	PPV (%)	NPV (%)
Foutch et al. [29]	1991	30	33	100	100	58
Ponchon et al. [30]	1995	204	35	97	96	44
Pugliese et al. [31]	1995	94	54	100	100	50
Mansfield et al. [32]	1997	43	42	100	100	8
Jailwala et al. [33]	2000	133	30	100	100	28
Stewart et al. [34]	2001	406	60	98	98	61
Kitajima et al. [35]	2007	60	72	100	100	32
Nishikawa et al. [36]	2014	123	51	98	97	63
Gerges et al. [37]	2020	27	21	100	100	65

PPV, positive predictive value; NPV, negative predictive value.

**Table 2:** The diagnostic yield of studies on forceps biopsy under fluoroscopic guidance.

Authors	Year	Number of Patients	Sensitivity (%)	Specificity (%)	PPV (%)	NPV (%)
Kubota et al. [39]	1993	41	81	100	100	75
Ponchon et al. [30]	1995	128	43	97	97	41
Sugiyama et al. [40]	1996	45	81	100	100	67
Jailwala et al. [33]	2000	133	43	90	94	31
Stewart et al. [34]	2001	406	60	98	98	61
Kitajima et al. [35]	2007	60	65	100	100	22
Hartman et al. [41]	2012	81	76	100	100	81
Nishikawa et al. [36]	2014	87	50	96	97	41

PPV, positive predictive value; NPV, negative predictive value.

**Table 3:** The diagnostic yield of studies on POCS visual findings and biopsy under direct view with POCS.

Authors	Year	Number of Patients	Visual Sensitivity (%)	Visual Specificity (%)	Visual Accuracy (%)	Biopsy Sensitivity (%)	Biopsy Specificity (%)	Biopsy Accuracy (%)
Chen et al. [48]	2007	22	100	77	85	71	100	90
Hartman et al. [41]	2012	89	88	86	87	57	100	78
Woo et al. [54]	2014	32	100	90	96	64	100	73
Kurihara et al. [55]	2016	89	94	92	94	65	89	70
Navaneethan et al. [49]	2016	44	90	96	N/A	85	100	N/A
Ogura et al. [56]	2017	25	83	89	93	80	100	89
Shah et al. [57]	2017	58	97	93	94	86	100	91
Gerges et al. [37]	2020	31	96	67	87	68	100	77
Tanisaka et al. [58]	2020	30	100	77	90	82	100	90

POCS, peroral cholangioscopy; N/A, not available.

**Table 4:** The diagnostic yield of studies on pCLE.

Authors	Year	Number of Patients	Sensitivity (%)	Specificity (%)	Accuracy (%)
Meining et al. [64]	2011	89	98	67	81
Meining et al. [65]	2012	45	97	33	N/A
Caillol et al. [66]	2013	89	96	64	78
Slivka et al. [67]	2015	112	89	71	82
Dubow et al. [68]	2018	97	83	93	90
Tanisaka et al. [58]	2020	30	94	92	93

pCLE, probe-based confocal laser endomicroscopy.

**Table 5:** The diagnostic yield of studies on EUS-FNA.

Authors	Year	Number of Patients	Sensitivity (%)	Specificity (%)	Accuracy (%)	Adverse Events, n
Fritscher-Ravens et al. [74]	2004	44	89	100	91	None
Rösch et al. [75]	2004	50	43	100	70	N/A
DeWitt et al. [76]	2006	24	77	100	79	None
Mohamadnejad et al. [71]	2011	81	73	N/A	N/A	1 (hemobilia)
Weilert et al. [77]	2014	51	94	100	94	None
Onda et al. [78]	2016	47	84	100	87	None

EUS-FNA, endoscopic ultrasound-guided fine-needle aspiration; N/A, not available.

## Data Availability

Data sharing not applicable.
